# Editorial: Plant-parasitic nematode and plant interaction under abiotic and biotic stresses

**DOI:** 10.3389/fpls.2023.1321382

**Published:** 2023-11-07

**Authors:** Jingsheng Chen, Xiaoli Guo, Guiping Yan, Yanfeng Hu

**Affiliations:** ^1^ College of Biology and Food Engineering, Chongqing Three Gorges University, Wanzhou, China; ^2^ College of Plant Science and Technology, Huazhong Agricultural University, Wuhan, China; ^3^ Department of Plant Pathology, North Dakota State University, Fargo, ND, United States; ^4^ Center for Agricultural Technology, Northeast Institute of Geography and Agroecology, Chinese Academy of Sciences (CAS), Harbin, China

**Keywords:** plant, plant-parasitic nematode, interaction, biotic stresses, molecular mechanism editorial on the research topic

This editorial summarizes the contributions to the Frontiers Research Topic “Plant-parasitic Nematode and Plant Interaction under Abiotic and Biotic Stresses”, published under the Plant Pathogen Interactions section of the journal Frontiers in Plant Science journal.

Editorial on the Research Topic

Not only do abiotic and biotic stressors affect plant development, growth, and productivity, but they also influence both the incidence and the severity of infection by plant pathogens. Plant-parasitic nematodes (PPNs) inflict significant biotic stress on numerous crops around the world, with over 4000 species threatening plant health and food security (Press and Phoenix, 2005) and resulting in an annual global loss of USD 173 billion (Elling, 2013). As a result, there is an increased focus on the influence of biotic and abiotic stressors on interactions between plants and nematodes, allowing the prediction of disease outbreaks, the breeding of better crop plants, and the design of effective means of control, especially in the context of climate change. Therefore, the aim of this Research Topic is to highlight recent research into the influence of biotic and abiotic factors on plant–nematode relationships.

Currently, PPNs in crops are best managed by the use of resistant cultivars. The breeding of such cultivars is dependent on an understanding of the molecular mechanisms associated with PPN resistance in plants. One article by Jiang et al. describes a novel avenue for the investigation of resistance to the soybean cyst nematode (*Heterodera glycines*, SCN) in soybean plants. The study identifies 10 novel QTLs and one QEI associated with 101 genes linked to SCN resistance using 3VmrMLM. The authors demonstrate the presence of a haplotype in Glyma.03G005600 that contributes to multi-SCN-race resistance and is linked to SCN HG Type 0 and Type 1.2.3.5.7 resistance. Another article that makes a direct contribution to plant breeding practice has also been contributed by Jiang et al. The *GmHg1* gene, encoding a serine/threonine protein kinase, was found to be associated with SCN resistance. According to the research, 11 genes co-expressed with *GmHg1* may contribute to soybean resistance to SCN. These findings suggest a novel strategy for facilitating the breeding of SCN-resistant plants.

The study by You et al. demonstrates the induction of the *CYP* gene *HgCYP33E1* by xenobiotics, such as pesticides, host plant metabolites, and biocontrol bacteria. The expression of *HgCYP33E1* is found to be influenced by SCN sensitivity to abamectin, indicating the participation of *HgCYP33E1* in xenobiotic biotransformation. Another article that makes a direct contribution to methylation-dependent modulation of the plant response to SCN infection is also included. Bennett et al. profile the activities of the promoters of 12 genes associated with DNA methylation and demethylation in Arabidopsis roots following infection by the beet cyst nematode (*Herodera schachtii*) and southern root-knot nematode (*Meloidogyne incognita*). They found that the promoter regions of several DNA demethylases show greater activity in galls compared with syncytia, especially during the initial stages of infection. Defective CG or CHH methylation was found to enhance plant susceptibility to both pathogens, while mutants with defective CHG methylation showed decreased susceptibility only to *M. incognita*.

Two other articles that make a direct contribution to biological control are included. Wang et al. investigated DEGs, DAMs, and lignin accumulation following infection by the root-knot nematode (RKN) in *Pnotoginseng notoginseng* plants of different ages using transcriptomics, metabolomics, and histochemistry. *P. notoginseng* was found to use a multipronged defensive approach to stop the growth and spread of RKN. This was accompanied by enhancement or inhibition of the flavonoid and phenylpropanoid pathways. In another article, Zhao et al. report that the highly virulent Trichoderma T1910 strain controls *M. incognita* infection in tomato, allowing further elucidation of the molecular mechanisms underlying the effects of Trichoderma spp. on *M. incognita*.

The articles presented in this Research Topic are valuable for the management of PPNs. These results will help nematode researchers, geneticists, and breeders to improve their understanding of interactions between plants and parasitic nematodes under conditions of biotic and abiotic stress.

## Author contributions

JC: Writing – original draft, Writing – review & editing. XG: Writing – review & editing. GY: Writing – review & editing. YH: Writing – original draft, Writing – review & editing.

## References

[B1] Elling A. A. (2013). Major emerging problems with minor meloidogyne species. Phytopathology 103, 1092–1102. doi: 10.1094/PHYTO-01-13-0019-RVW 23777404

[B2] Press M. Phoenix G. (2005). Impacts of parasitic plants on natural communities: Tansley review. New Phytol. 166, 737–751. doi: 10.1111/j.1469-8137.2005.01358.x 15869638

